# Registered Report: Transcriptional Analysis of Savings Memory Suggests Forgetting is Due to Retrieval Failure

**DOI:** 10.1523/ENEURO.0313-19.2020

**Published:** 2020-11-12

**Authors:** Tania Rosiles, Melissa Nguyen, Monica Duron, Annette Garcia, George Garcia, Hannah Gordon, Lorena Juarez, Irina E. Calin-Jageman, Robert J. Calin-Jageman

**Affiliations:** Neuroscience Program, Dominican University, River Forest, Illinois 60305

**Keywords:** latent memory, long-term memory, sensitization, transcription

## Abstract

There is fundamental debate about the nature of forgetting: some have argued that it represents the decay of the memory trace, others that the memory trace persists but becomes inaccessible because of retrieval failure. These different accounts of forgetting lead to different predictions about savings memory, the rapid re-learning of seemingly forgotten information. If forgetting is because of decay, then savings requires re-encoding and should thus involve the same mechanisms as initial learning. If forgetting is because of retrieval failure, then savings should be mechanistically distinct from encoding. In this registered report, we conducted a preregistered and rigorous test between these accounts of forgetting. Specifically, we used microarray to characterize the transcriptional correlates of a new memory (1 d after training), a forgotten memory (8 d after training), and a savings memory (8 d after training but with a reminder on day 7 to evoke a long-term savings memory) for sensitization in *Aplysia californica* (*n *=* *8 samples/group). We found that the reactivation of sensitization during savings does not involve a substantial transcriptional response. Thus, savings is transcriptionally distinct relative to a newer (1-d-old) memory, with no coregulated transcripts, negligible similarity in regulation-ranked ordering of transcripts, and a negligible correlation in training-induced changes in gene expression (*r *=* *0.04 95% confidence interval (CI) [–0.12, 0.20]). Overall, our results suggest that forgetting of sensitization memory represents retrieval failure.

## Significance Statement

Understanding the nature of forgetting is important because both excessive and insufficient forgetting are related to profound disruptions of mental health. This registered report provides molecular data indicating that forgetting of long-term sensitization in *Aplysia* represents retrieval failure, contributing new evidence toward resolving a long-standing debate over the neural mechanisms of forgetting.

## Introduction

Long-term memory is characterized both by its duration and by its dependence on changes in gene expression (Goelet et al., 1986). Although long-term memories can last a lifetime, much of what we initially commit to long-term memory is forgotten, becoming progressively less likely to be recalled (Bahrick, 1984). Forgetting plays an essential role in memory function as both excessive and insufficient forgetting are related to profound disruptions of mental health (Tröster et al., 1993; Ally et al., 2013; Fitzgerald et al., 2013; Mary et al., 2013).

Currently, there is fundamental disagreement about the nature of forgetting, with one review concluding that “we do not know why or how the brain actually forgets” (p 113, Hardt et al., 2013). Some have argued that forgetting occurs because of decay of the memory trace and, thus, represents a failure of memory maintenance. In stark contrast, others have suggested that forgetting is merely a retrieval failure, and that the original memory trace persists, perhaps indefinitely (for review, see Wixted, 2004; Davis and Zhong, 2017). For example, forgetting could be because of inhibitory processes that repress otherwise intact memory traces (Barron et al., 2017).

Here, we conduct an experiment to shed light on the nature of forgetting by studying savings memory, the rapid re-acquisition of seemingly forgotten information. Ebbinghaus first characterized savings memory (Ebbinghaus, 1885). He learned lists of nonsense words to perfection, waited until he could no longer recall the words, and then re-learned the lists to perfection. He found that it always took less training to re-learn the lists compared with the original acquisition. Since that pioneering demonstration, savings memory has been demonstrated with multiple learning paradigms (Nelson, 1985) and in a variety of species (Antzoulatos et al., 2006; Philips et al., 2006; Menges et al., 2015), suggesting that it is a core feature of long-term memory.

**Figure 1.**
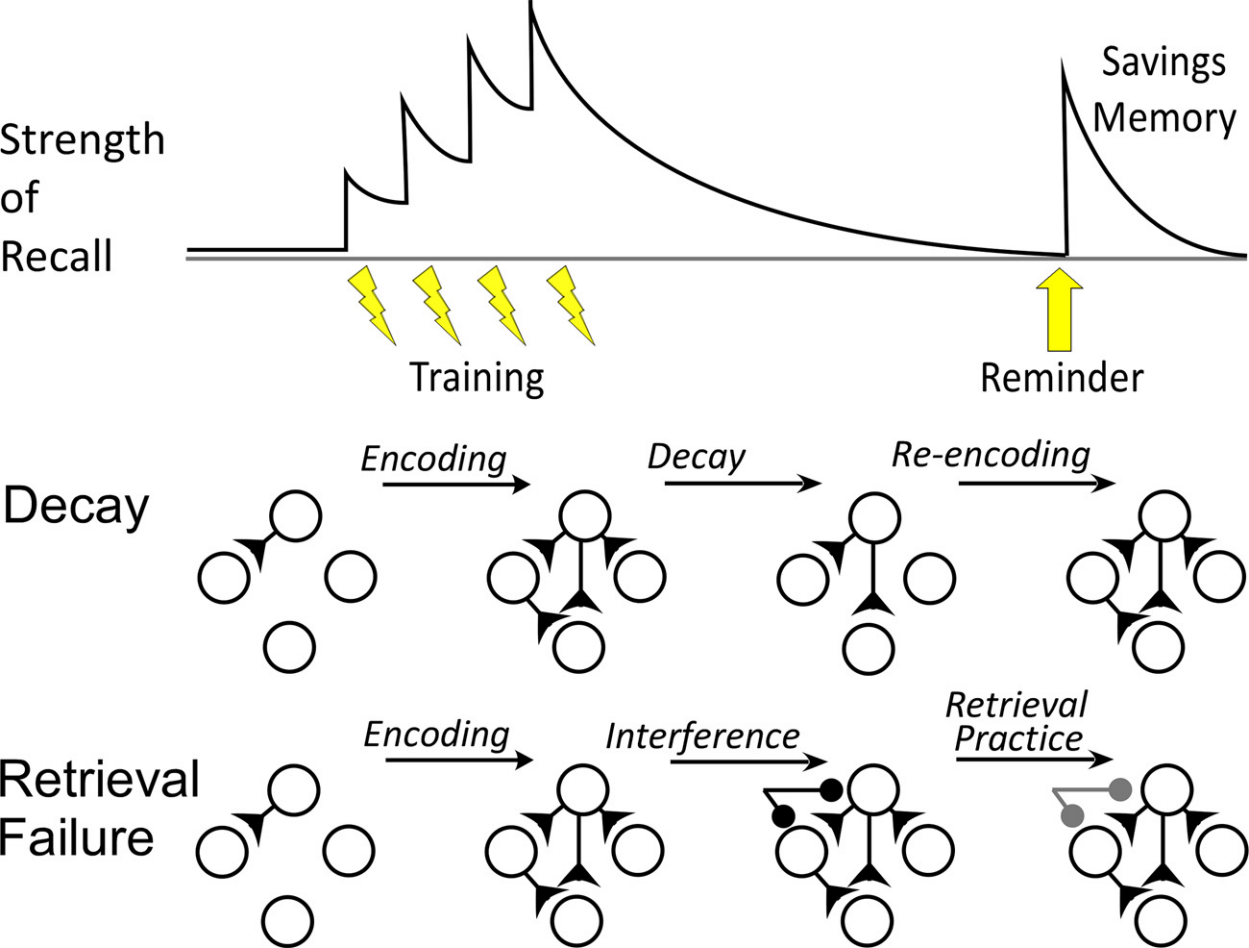
Savings memory according to decay and retrieval-failure accounts of forgetting. Top, Savings memory. Repeated rounds of training (lightning bolts) increase strength of recall, but in the absence of additional practice, forgetting occurs, indicated by a decline in the strength of recall toward zero. Nevertheless, a brief reminder can re-instate recall; this is known as savings memory. Middle, In decay theories of forgetting, initial learning changes synaptic connectivity and strength forming a memory trace. Over time, however, these changes decay away, leading to forgetting (reduced recall). During savings memory, the memory trace must be almost entirely rebuilt. Savings is thus predicted to use the same transcriptional mechanisms that initially created the memory trace. Bottom, In retrieval-failure theories of forgetting, forgetting is due not to decay but to interference from other memories. For example, additional learning could inhibit (dark circles) the otherwise intact memory trace. In this framework, savings involves repairing retrieval mechanisms (e.g., downregulating inhibition). Thus, savings is predicted to be transcriptionally distinct from initial memory storage.

Decay-failure and retrieval-failure accounts of forgetting make contrasting predictions about the transcriptional correlates of savings memory (Fig. 1). If memory traces decay, then savings is a re-construction of the original information facilitated by the remnants of the original memory trace. Under this account, savings is predicted to be mechanistically similar to initial memory storage and should evoke a transcriptional state similar to what is observed during new learning. If, on the other hand, forgetting involves only retrieval failure, then savings does not require rebuilding the original memory trace. In this case, savings memory would be mechanistically and transcriptionally distinct from initial memory storage.

**Figure 2.**
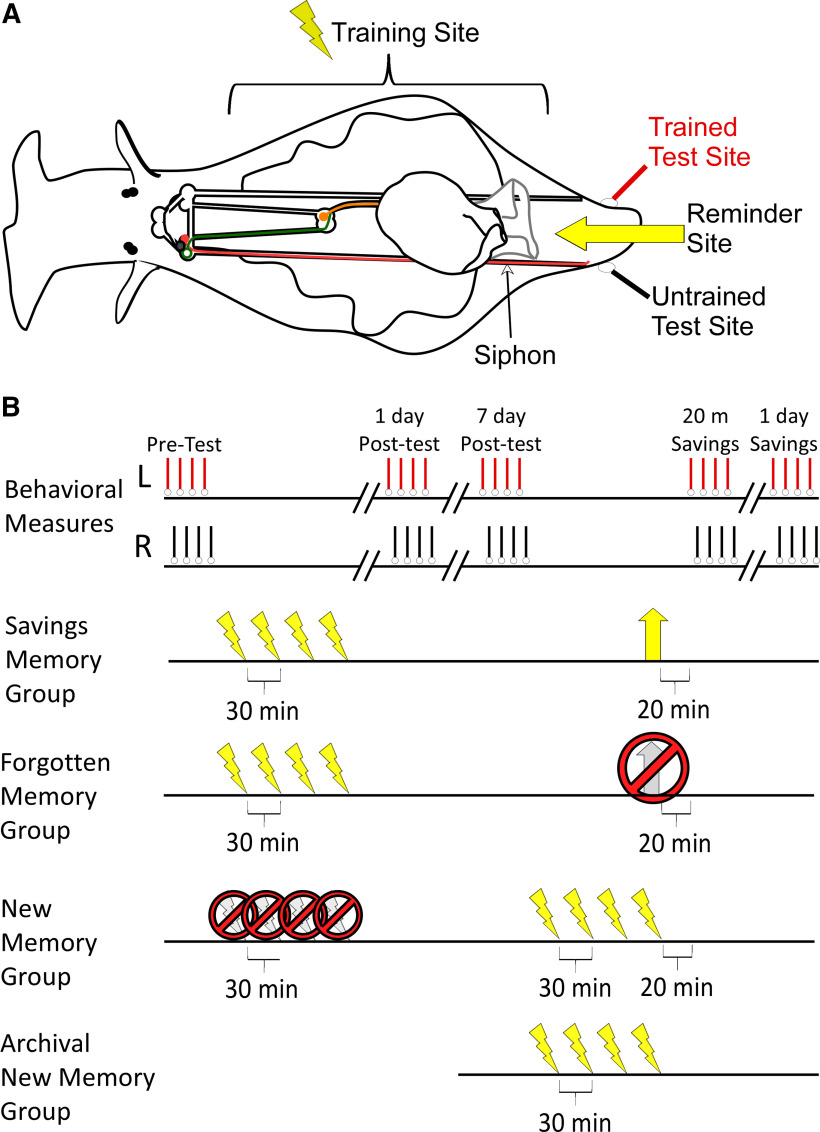
Long-term sensitization in *Aplysia*. ***A***, Overview of the behavioral paradigm. Sensitization is an increase in responsiveness because of noxious stimulation. To produce long-term sensitization in *Aplysia*, animals were exposed to four rounds of painful shock to one side of the body (training site: lightning bolt). The effect of training was monitored by measuring the duration of the T-SWR, a defensive withdrawal of the siphon evoked by an innocuous stimulus to the left or right side of the tail (test site). To document savings, a reminder was administered to the midline of the tail (reminder site, wide arrow). In naive animals this reminder produces short-term but not long-term sensitization. ***B***, Behavioral protocols. Behavioral measures were the same in all experimental groups: T-SWR responses were evoked on the left and right tail (red and black hash lines). Responses were measured at pre-test, 1 d after training, 7 d after training, 20 min after the reminder, and 1 d after the reminder (day 8 from training). Animals differed in their experimental treatments. For the savings-memory group, animals received standard sensitization training after pre-test measures (four strong shocks, 30 min apart, lightning bolts). Then, 7 d after training animals received the reminder (wide arrow) to evoke savings memory. For the forgotten-memory group, the treatment was the same except animals were given sham reminder (gray arrow crossed out), leaving the sensitization memory dormant. Finally, for new-memory group, animals initially received sham sensitization training (gray lightning bolts crossed out) and then received real sensitization training after the 7-d post-tests. All animals were harvested for microarray analysis after the 1-d savings tests (day 8 from start). Thus, all animals received the same behavioral testing but, when harvested, were expressing different states of sensitization memory: new (1 d since training), forgotten (8 d since training with no reminder), or savings (8 d since training and 1 d since the reminder). In addition, we analyzed an archival dataset from Conte et al. (2017), which roughly replicates the new-memory group (harvested 1 d after sensitization training). We used these archival data to benchmark assessments of similarity.

In this registered report, we tested the decay-failure and retrieval-failure accounts of savings memory (Fig. 2). Specifically, we used microarray to characterize the transcriptional changes that accompany long-term sensitization in *Aplysia californica* for a newly stored memory (1 d after training), a forgotten memory (8 d after training), and a savings memory (8 d after training but with a reminder on day 7).

Long-term sensitization in *Aplysia* requires changes in transcription (Sutton et al., 2001), and new memory storage is associated with regulation of over 1000 transcripts within 1 d of training (Conte et al., 2017). As sensitization memory is forgotten most of this transcriptional response fades; 7 d after training only approximately seven transcripts remain regulated (Patel et al., 2018; Perez et al., 2018). Here, we asked whether savings memory reactivates the transcriptional changes observed with new memory formation (as predicted if forgetting is because of decay) or if savings has a distinct transcriptional profile (as predicted if forgetting is because of retrieval failure).

Comparing transcriptional states with microarray can be difficult because poor signal-to-noise and sampling error can produce spurious dissimilarity. These problems can be overcome in the learning paradigm we selected. First, sensitization-related transcription in *Aplysia* can be characterized from isolated ganglia that contain neurons known to help encode sensitization memory, providing a strong learning-related transcriptional signal (Herdegen et al., 2014a). Second, learning is expressed on only the trained side of the body (Scholz and Byrne, 1987), enabling a powerful within-subjects comparison. Thus, microarray experiments of moderate size (eight samples/group) can attain high power and convergent validity (Herdegen et al., 2014b). Third, we have previously analyzed the transcriptional correlates of newly stored sensitization memory (1 d after training), providing a set of benchmarks for analyzing the correlates of savings memory (Conte et al., 2017).

Leveraging the advantages of the sensitization paradigm we conducted a rigorous experiment to provide compelling evidence about the nature of forgetting. We found that the transcriptional correlates of savings memory are distinct from new memory formation suggesting that forgetting of sensitization is because of retrieval failure.

## Materials and Methods

We conducted this study as a registered report. First, we developed and publicly posted a behavioral protocol, quality controls, and a behavioral analysis plan (https://osf.io/z2uck; May 28, 2018). Then, we began initial collection of behavioral data and sample preparation. Once we had enough behavioral data to be confident our design was feasible, we developed a microarray analysis plan and script and submitted a registered report proposal. After review and in-principle acceptance we publicly preregistered the study (https://osf.io/fqh8j; September 11, 2019), completed behavioral data collection, and conducted the planned microarray analysis.

### Open data and materials

Our preregistration, analysis scripts, and all data for this project are available on the Open Science Framework (https://osf.io/z2uck/). The microarray data are also posted to NCBI’s Gene Expression Omnibus (GEO: GSE152045, https://www.ncbi.nlm.nih.gov/geo/query/acc.cgi?acc=GSE152045).

### Animals

Animals (75–125 g) were obtained from the RSMAS National Resource for *Aplysia* and maintained at 16°C in one of two 90-gallon aquariums with continuously circulating artificial sea water (Instant Ocean, Aquarium Systems Inc.).

### Long-term sensitization training

A 1-d long-term sensitization training protocol (Fig. 2) was used (Bonnick et al., 2012). Training consisted of four rounds of noxious shock applied at 30-min intervals to one side of the body with a hand-held electrode. Each round of shock consisted of 10 pulses (60-Hz biphasic) of 500-ms duration at a rate of 1 Hz and an amplitude of 90 mA. Side of training was counterbalanced. This training protocol produces memory that is strongly expressed for several days but which fades in most animals within one week (Perez et al., 2018).

The savings-memory and forgotten-memory groups received long-term sensitization training immediately after pre-tests, on the first day of the protocol. In contrast, the new-memory group initially received sham training. This consisted of the same procedure but the constant-current stimulator was set to deliver 0 mA of current. Animals were otherwise handled in the same way and, in general, were run mixed with batches of animals from the other conditions. For the new-memory group, real sensitization training was finally applied after the 7-d tests, 1 d before harvesting tissue.

### Reminders to elicit savings

To elicit savings, animals in the savings group received a reminder shock (Philips et al., 2006; Perez et al., 2018). The reminder was delivered 7 d after training, when most animals show essentially no remaining sensitization memory (<25% increase relative to pre-test). The reminder consisted of two moderate shocks (60-Hz biphasic DC pulse for 2 s at 20 mA of constant current) applied to the midline of the tail with a 15-min rest between the shocks. The reminder produces short-term but not long-term sensitization in naive animals. In previously trained animals the reminder reveals a long-lasting unilateral savings memory, with tail-elicited siphon-withdrawal reflex (T-SWR) durations increasing for at least 1 d after the reminder but only on the previously trained side (Perez et al., 2018).

Animals in the forgotten-memory group received a sham reminder, where the same protocol was applied, but the constant-current stimulator was dialed to deliver 0 mA of current.

Animals in the new-memory group did not receive a reminder or a sham reminder but, instead, received their sensitization training while the other groups received reminders.

### Behavioral measurement

As a behavioral outcome, we measured the duration of the T-SWR (see Walters and Erickson, 1986). The reflex was evoked by applying a weak shock to one side of the tail using a hand-held stimulator (60-Hz biphasic DC pulse for 500 ms at 2 mA of constant current). T-SWR behavior was measured as the duration of withdrawal from the moment of stimulation to the first sign of siphon relaxation.

Measurements were made blind to experimental condition. For each time point (pre-test, 1-d, 7-d, 20-min savings, and 1-d savings) behavioral responsiveness was characterized by a series of eight responses evoked on alternating sides of the body at a 10-min ISI. Scores were split by side of stimulation (trained vs untrained) and averaged (four responses/side for each time point characterized).

### Isolation and processing of pleural ganglia RNA

We compared gene expression from pleural ganglia on the trained versus untrained side of the animal. The pleural ganglia contain the ventro-caudal (VC) nociceptors (Walters et al., 2004) which contribute input to the T-SWR circuit as well as several T-SWR interneurons (Mackey et al., 1987; Buonomano et al., 1992; Cleary and Byrne, 1993). The VCs are essential for encoding long-term sensitization memories. Gene expression measured in whole pleural ganglia correlates strongly with expression measured from isolated VC clusters (Conte et al., 2017).

To control for lateralized gene expression, samples from two animals trained on opposite sides were pooled.

To analyze transcription, pleural ganglia RNA were isolated immediately after the long-term savings test, 8 d after protocol start. Animals were anesthetized with an injection of isotonic MgCl_2_ (50% of body weight), and an incision was then made along the ventral midline to expose the CNS. As dissection can alter gene expression (Alberini et al., 1994), we extracted ganglia rapidly (<5 min per animal) and transferred them immediately to Trizol (Invitrogen) for homogenization.

Tissue was homogenized using the Bullet Blender (NextAdvance) and RNA extracted using Direct-Zol Mini RNA kit (Zymo). Quantity and quality of RNA was assessed using the NanoDrop 1000 (Thermo Scientific).

### Reverse-transcription quantitative PCR (qPCR)

Reverse transcription was performed using Maxima cDNA kit with DsDNase (Thermo Scientific). Quantitative PCR was conducted using Maxima SYBR Green/Fluorescein qPCR Master Mix (Thermo Scientific) and the MyIQ real time PCR system (Bio-Rad). Primers were validated for correct PCR efficiency; exact sequences are provided in Patel et al. (2018), their Supplemental Table 1. qPCR samples were analyzed in duplicate or triplicate and the relative amounts of each transcript were determined using the ddCT method and the Bio-rad IQ5 gene expression analysis. All qPCR expression levels were normalized to levels of histone H4, a transcript which is stable during LTS training.

### Sample size determination

We set a target of eight biological replicates per group. This sample-size exceeds the consensus recommendation of at least five biological replicates per group for microarray analysis (Pavlidis et al., 2003; Tsai et al., 2003; Allison et al., 2006). Moreover, previous transcriptional analyses (Herdegen et al., 2014b) using this learning paradigm has shown that 8 samples per group can achieve very low estimated false-positive rates (1–2%) and strong convergent validity with qPCR conducted in independent samples (*r*^2^ = 0.60–0.79). Finally, we tested our analysis script with real data of known levels of similarity and found that this sample-size was sufficient to distinguish between highly similar, moderately similar, and orthogonal sets of regulated transcripts.

### Archival data to benchmark similarity

Our microarray analyses compared gene expression in the new-memory, forgotten-memory, and savings-memory groups. To help provide context for these comparisons, we also re-analyzed gene expression from a previous study examining transcriptional changes 1 d after long-term sensitization (Conte et al., 2017; GEO: GSE95596). We refer to this as the “archival new-memory group.” These archival data essentially replicate the new-memory group, although they did not involve as many rounds of T-SWR measurement nor sham training (see Fig. 2).

### Microarray processing

We used the Aplysia Tellabs Array (ATA; GEO: GPL18666) to characterize changes in gene expression because of long-term sensitization training (Herdegen et al., 2014b). This array includes 26,149 distinct probes representing all known sources of *A. californica* ESTs and mRNAs at the time of design (January 2012). Based on estimates from previous microarray designs (Moroz et al., 2006), the ATA should cover >50–60% of all CNS-expressed transcripts.

Microarray processing was completed by Mogene Inc. A two-color approach was used, with each array hybridized to a paired trained and untrained sample. Specifically, each of the 24 arrays compared expression from the trained side of a left-trained and right-trained animal to expression from the untrained sides of the same animals. Experimental condition (savings, new-memory, forgotten-memory) was balanced across slides (*n* = 8/group). In addition, dye color was counterbalanced across training conditions.

Sample integrity was determined by Bioanalyzer RNA 6000, Pico total RNA protocol; 300 ng of total RNA was amplified and labeled with Cy3 or Cy5 using the Agilent Quick Amp Two-Color Labeling kit. Dye incorporation and yield was determined by Nanodrop. Samples were hybridized to the microarray slide at 65°C and 10 rpm for 17 h. Slides were scanned on an Agilent C scanner at 3- μm resolution. Data were extracted using Agilent Feature Extraction software, version 11.5. All labeling and postlabeling processing was conducted in an ozone regulated environment, monitored at <5 ppb.

### Statistical analyses

In our statistical analyses we focus on effect sizes and 95% confidence intervals (CIs; Calin-Jageman and Cumming, 2019a,b). These can easily be converted to hypothesis tests. If the null hypothesis is not in the 95% CI, the test is significant at α = 0.05; otherwise the test is not significant.

### Behavioral analysis

Behavioral responses were averaged by time point and side of testing (trained or untrained). Change scores were then calculated by subtracting pre-test scores. At each time point paired comparisons were made between the average change on the trained side and the average change on the untrained side: *M*_diff_ = (*M*_trained_change_ − *M*_untrained_change_). The 95% CI for this contrast was then calculated. This is equivalent to estimating the interaction between training and time with a 2 × 2 within-subjects ANOVA. Along with raw-score effect sizes we report standardized effect size estimates (Cohen’s *d*). These are corrected for bias (Hedges, 1981) and calculated so that positive values represents a stronger increase in response on the trained side (sensitization).

### Microarray

Microarray data were analyzed using limma (Smyth, 2005; Ritchie et al., 2015) from the Bioconductor suite of tools (Gentleman et al., 2004) for R (Ihaka and Gentleman, 1996). Median expression values were analyzed (Zahurak et al., 2007). These were corrected for background using the normexp+offset algorithm recommended for Agilent microarrays by Ritchie et al. (2007). Expression was then normalized using the loess function (Smyth and Speed, 2003). Where multiple probes were used to measure the same EST or mRNA, these were averaged (Holmes et al., 2014).

### Identification of regulated transcripts

Within each experimental group, trained and control expression was compared by computing a log-fold change (LFC) score indicating the ratio of expression from the trained to control sides (base 2). Changes in expression were flagged by using the treat function from limma (McCarthy and Smyth, 2009) to test for regulation significantly greater than 10% in either direction (an interval null from −10% to 10%) with an empirical Bayes-moderated *t* test (Smyth, 2004). Benjamini–Hochberg correction was used to maintain a 5% overall false discovery rate (Benjamini and Hochberg, 1995). All the CIs reported for individual transcripts reflect the same correction for multiple comparisons.

### Check for completeness of gene lists

We estimated the proportion of true nulls in each condition using the propTrueNull function (Ritchie et al., 2015) using the convex decreasing densities approach developed by Langaas et al. (2005). We then calculated the false negative rate as 1 – %regulated – %truenull. Based on previous analyses we established a criterion of false negative rates <4% for subsequent comparisons of transcription to be considered valid.

### Degree of overlapping regulation

For the genes regulated in the new-memory group (1 d after training) we examined the proportion (*P*) also regulated in the forgotten-memory, savings-memory, and archival new-memory groups. We then estimated the difference in overlap as a memory is reactivated during savings: *P*_diff_ = *P*_savings_overlap_ – *P*_forgotten_overlap_.

To follow-up on this analysis of overlap we tested formally for differences in regulation between the forgotten-memory and savings-memory groups among the transcripts regulated 1 d after training. This is equivalent to testing each transcript for a training × condition interaction. We again used an interval null of ±10%.

### Similarity of ranked transcript lists

We used the OrderedList package for R (Yang et al., 2019) to calculate similarity scores based on overlap across ordered ranks of transcripts. For each condition, transcripts were first ranked by strength of evidence (*p* value) and sign of regulation (up or down). We then computed and assessed overlap across the top and bottom ∼1000 transcripts in each list.

### Correlations in training-evoked expression

We also examined correlations in LFC scores. The critical question to examine was similarity to the new-memory group, so we first restricted down to the transcripts flagged as clearly regulated in this group. Then we calculated the correlation in LFC to the savings (*r*_new_savings_), forgotten-memory (*r*_new_forgotten_), and archival new-memory groups (*r*_new_archivalnew_). We calculated each relationship with a simple Pearson’s *r* and with correction for potential measurement error using the genuine association of gene-expression profiles function (genas) in limma (Ritchie et al., 2015).

As our primary outcome we examined whether the savings-memory and forgotten-memory groups differed in similarity to the new-memory group. To do this we calculated the difference in correlations across these groups (*r*_diff_ *= r*_new_forgotten_ – *r*_new_savings_) using the paired.r function from the psych package in R (Revelle, 2018). We expected that if savings reactivates transcriptional mechanisms of memory storage then it should show stronger similarity to the new-memory group, leading to a positive value of *r*_diff_. We pre-established the following interpretations based on analysis of previous data: strong increase in similarity if *r*_diff._ ≥ 0.5, moderate increase in similarity if *r*_diff._ ≥ 0.25 but <0.50, little-to-no increase in similarity if *r*_diff._ < 0.25.

### Data collection and quality controls

We collected data from 98 *Aplysia*. With pairing left-trained and right-trained animals this provided 49 RNA samples (15 assigned to the savings-memory group, 16 in the new-memory group, and 18 in the forgotten-memory group). Making a fair test between decay and retrieval-failure accounts of forgetting requires that the transcriptional analysis proceeds with samples that exemplify each memory state. Therefore, before data collection, we established a strong set of quality controls and posted them publicly to the Open Science Framework (https://osf.io/z2uck/wiki/Experimental%20Protocol/; posted on May 28, 2018).

First, we checked behavioral data to ensure each sample selected for microarray exemplified the desired memory state:

(1) To ensure training effectiveness we required all animals to show robust but unilateral long-term sensitization on measures taken 1 d after their training (>30% increase in T-SWR duration on the side of training and less than a 30% change on the untrained side). All but two animals met this criterion; the samples they were part of were discarded (one from the savings-memory group, one from the forgotten-memory group).

(2) There is some variation in *Aplysia* in forgetting. To ensure that the savings and forgotten-memory groups truly represent a forgotten-memory state we required that animals in these groups show negligible behavioral sensitization by the 7-d tests, indicated by T-SWR durations that have returned to within 25% of pre-test. Two animals from the forgotten-memory group did not meet this criterion (they showed lingering sensitization), so the two samples they were part of were discarded.

(3) We required animals in the savings group to show savings memory after the reminder, defined as having T-SWR durations during the savings test that had increased over baseline more on the previously-trained side than on the previously-untrained side. All animals assigned to the savings group met this criterion.

(4) We checked to ensure that habituation from repeated testing did not contaminate our comparison, requiring that T-SWR measures on the 7-d test were within 30% of baseline on the untrained side. Animals from one sample in the forgetting condition did not meet this criterion. However, in coding this criterion we accidentally applied it to the trained side (which all samples passed) and this error was not detected until after this sample had been included in the microarray analysis. As reported in the exploratory analysis section, excluding this sample did not impact the results of the study.

We also checked the quantity and quality of isolated RNA for each sample, discarding any samples with a very low or uneven yield, poor quality, and/or genomic contamination. Based on this, we discarded an additional 12 samples (six from the new-memory group and six from the forgotten-memory group).

Finally, to ensure samples had been properly processed, we used quantitative real-time PCR to check for upregulation of well-defined transcriptional markers of sensitization training:

(1) For the new-memory group, we checked for upregulation of the transcript encoding ApBiP (GenBank: NM_001204652; Kuhl et al., 1992). This transcript is strongly and consistently upregulated 1 d after sensitization training (Conte et al., 2017). As expected, there was strong upregulation of ApBiP in samples from the new-memory group (LFC = 1.49 95% CI [0.79, 2.18], *n* = 10). However, one sample unexpectedly showed lower expression on the trained side. We discarded this sample.

(2) We have not previously examined transcriptional correlates of savings, but we reasoned that transcripts which remain persistently regulated during forgetting should still be regulated during savings. Thus, we checked the savings-memory group for regulation of the transcript encoding the peptide neurotransmitter Phe-Met-Arg-Phe NH2 (FMRFa; GenBank: M11283.1; Schaefer et al., 1985). This transcript is strongly upregulated within 1 d of sensitization training and continues to be upregulated for more than one week (Patel et al., 2018). As expected, we observed strong upregulation of FMRFa in this group (LFC = 1.28 95% CI [0.86, 1.69], *n* = 14). We found two samples, however, with decreased FMRFa expression on the trained side; these were discarded.

(3) We did not specify a positive control for the forgotten-memory group, but as expected, there was upregulation of FMRFa in this group (LFC = 0.61 95% CI [0.01, 1.21], *n* = 9).

For each group we selected eight valid samples, leaving one to four samples per group for potential use with qPCR validation.

## Results

First, we report the behavioral data, which were collected primarily before preregistration to ensure the samples would adequately represent the different stages of memory. Then, we report our preregistered microarray analysis; all and only planned analyses are reported. Finally, we report additional exploratory analyses that were not part of our preregistered plan.

### Behavioral validation

**Figure 3.**
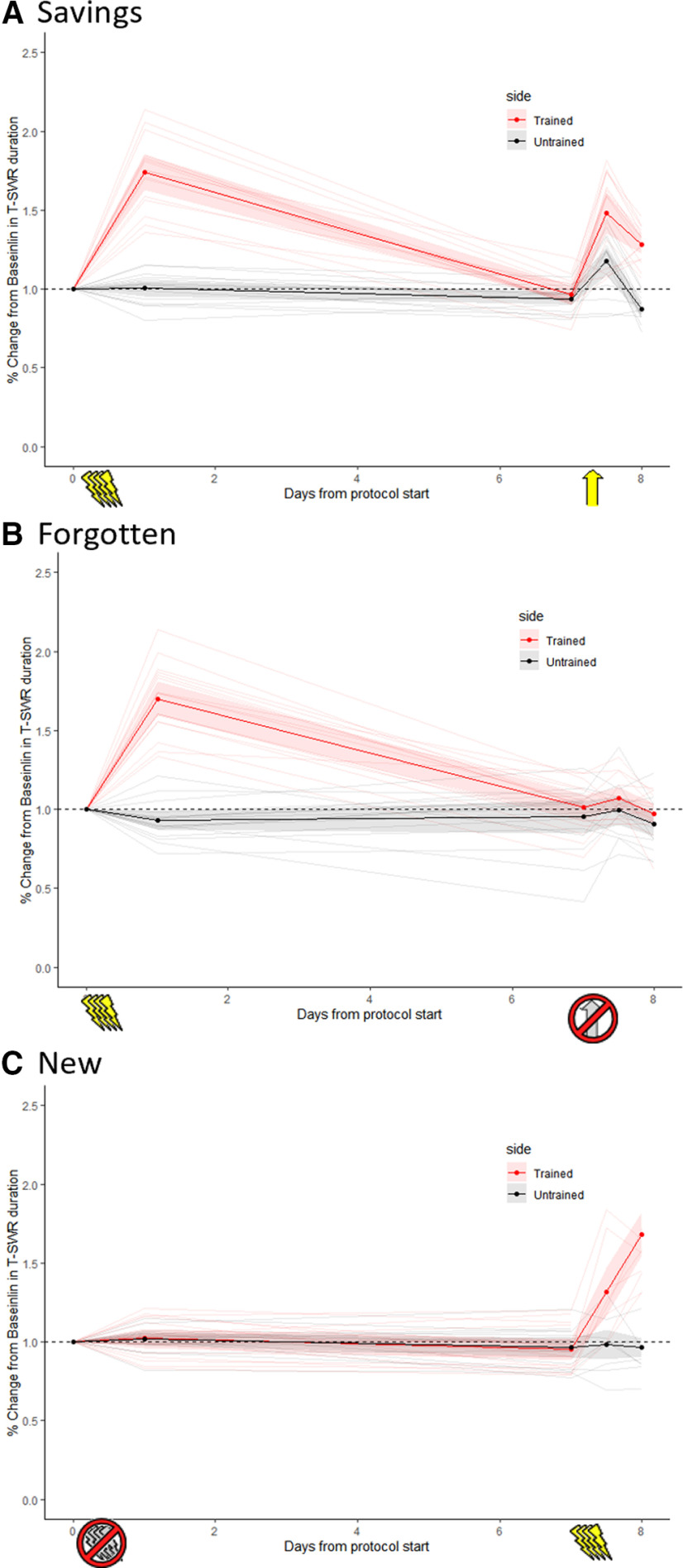
Behavioral changes in the savings, forgetting, and new-memory conditions. This figure shows T-SWR duration as a % change from pre-test on both the trained (red) and untrained (black) sides. Dark lines with dots represent group means; shading indicates 95% CI of the mean. Individual animals are represented by the light lines. ***A***, Savings-memory group, which received real training (lightning bolts) after pre-tests and a reminder (yellow arrow) after the 7-d tests. All animals were expressing a long-term savings memory when harvested on day 8. ***B***, Forgotten-memory group, which received real training after pre-tests but a sham reminder (crossed-out arrow) after the 7-d tests. All animals showed apparent forgetting when harvested. ***C***, New-memory group, which received sham training (crossed-out lightning bolts) after pre-tests and then real training after the 7-d tests. All animals were expressing a new (1-d-old) memory when harvested.

We confirmed that samples selected for microarray showed the expected trends in sensitization memory. In the savings group (*n *=* *16 animals to provide 8 samples; Fig. 3*A*), training produced long-term sensitization, expressed as a large but unilateral increase in T-SWR duration when tested 1 d later. T-SWR responses increased by 5.4 s on the trained side (95% CI [4.7, 6.1]) but showed no change on the untrained side (*M*_untrained_change_ = 0.0 s 95% CI [−0.4, 0.4]). Thus, comparing changes on the trained and untrained side indicated a very large training effect (*M*_diff_ = 5.4s 95% CI [4.7, 6.1], *d *=* *4.7 95% CI [3.7, 6.5]). Although initial learning was strong, sensitization was then forgotten, as by the 7-d post-tests responses were slightly below pre-test on both sides (*M*_trained_change_ = −0.2 s 95% CI [−0.7, 0.2]; *M*_untrained_change_ = −0.6s 95% CI [−1.0, −0.2]), so there was only a weak residual training effect (*M*_diff_ = 0.3s 95% CI [−0.2, 0.9], *d *=* *0.4 95% CI [−0.2, 1.1]). Despite this apparent forgetting, all animals showed robust savings memory, as a reminder evoked a long-term re-expression of sensitization on the previously untrained side. Specifically, 1 d after the reminder (1-d savings test) T-SWR responses were moderately increased on the previously trained side (*M*_trained_change_ = 2.0 s 95% CI [1.7, 2.4]) but continued to be slightly below pre-test on the previously untrained side; *M*_untrained_change_ = −1.0 s 95% CI [−1.3, −0.6]), reinstating a relatively large training effect indicative of savings memory (*M*_diff_ = 3.0 s 95% CI [2.6, 3.5], *d *=* *4.3 95% CI [3.3, 6.0]). Savings memory was also evident when normalized to 7-d post-test scores (*M*_diff_ = 1.9 s 95% CI [1.2, 2.5], *d *=* *1.3 95% CI [0.9, 1.8]).

In the forgotten-memory group (*n *=* *16 animals to provide 8 samples; Fig. 3*B*), sensitization training also produced robust but unilateral increases in T-SWR duration (1-d post-tests: *M*_diff_ = 7.2 s 95% CI [6.0, 8.5], *d *=* *3.1 95% CI [2.3, 4.2]). Sensitization was largely, but perhaps not completely, forgotten within one week (7-d post-tests: *M*_diff_ = 0.9 s 95% CI [−0.1, 1.9], *d *=* *0.4 95% CI [0.0, 0.8]). The sham reminder did not produce a lasting change in behavior; long-term savings tests showed only weak behavioral expression of sensitization memory (*M*_diff_ = 0.8s 95% CI [0.0, 1.6], *d *=* *0.5 95% CI [0.0, 1.1]).

Finally, in the new-memory group (*n *=* *16 animals to provide 8 samples; Fig. 3*C*), sham training did not alter behavior, so there was no training effect at the 1-d post-tests (*M*_diff_ = 0.0 s 95% CI [−0.7, 0.7], *d *=* *0.0 95% CI [−0.7, 0.7]) nor at the 7-d post-tests (*M*_diff_ = 0.0 s 95% CI [−0.6, 0.6], *d *=* *0.0 95% CI [−0.5, 0.4]). Real sensitization training administered after the 7-d post-tests produced the expected unilateral sensitization, with a large training effect on measures taken the next day (savings post-test: *M*_diff_ = 6.4 95% CI [5.1, 7.6], *d *=* *3.3 95% CI [2.5, 4.6]).

Overall, animals selected for microarray exhibited clear and consistent behavioral patterns representative of new, forgotten, and savings stages of sensitization memory.

### Planned microarray analysis

The decay account of forgetting predicts that savings recapitulates most of the transcriptional response required to store a memory (savings will be similar to the new-memory group). The retrieval-failure account of forgetting predicts that savings will have distinct transcriptional mechanisms (savings will not be similar to the new-memory group). To test these predictions, we measured the similarity of microarray results between the groups. We assessed similarity in three different ways: (1) as the degree of overlap among transcripts flagged as regulated, (2) as the consistency of rank-order gene lists, and (3) as the linear correlation between LFCs in expression.

### How similar is savings memory to new memory? Overlapping regulation approach

As an initial way to characterize similarity we flagged clearly regulated transcripts at each epoch of memory (new, forgotten, and savings) and then calculated the overlap in flagged transcripts across conditions. We defined clearly regulated transcripts as those that showed significantly more than a 10% change in expression, with adjustment to maintain a 5% overall false discovery rate. This is a stringent criterion likely to miss some regulated transcripts, but our goal for this initial analysis was to compare results without noise from potentially negligible changes in expression (see next 3 sections for more sensitive comparisons and also for exploratory analyses based on less stringent criteria). Table 1 gives the counts of transcripts flagged in each group. Extended Data Table 1-1 gives full results for each transcript in each condition and is also posted to the Open Science Framework (https://osf.io/z2uck/).

**Table 1:** Counts of clearly regulated transcripts and overlap of regulation

Group	Upregulated	Downregulated	Estimated false negative rate	Proportion of new memory transcripts coregulated
New memory	131	17	<1%	
Forgotten memory	0	0	<1%	0.00 95% CI [0.00, 0.03]
Savings memory	0	0	<1%	0.00 95% CI [0.00, 0.03]
Archival new memory	798	463	<1%	0.95 95% CI [0.91, 0.98]

A complete table of microarray results is provided in Extended Data Table 1-1.

10.1523/ENEURO.0313-19.2020.t1-1 Extended Data Table 1-1 Complete table of microarray results. This table provides microarray results for each transcript in each condition. The Transcript column provides the unique identified for the transcript probe. The BestAnnotation column provides an annotation (if available) for that transcript. The Previous_finding column indicates if that transcript was previously identified as regulated after long-term sensitization training. The LFC columns report the mean LFC for that transcript (trained vs control) by condition (d1 for new, w1 for forgetting, sav for savings). The adj.MoE columns report the 95% margins of error for these mean LFCs, adjusted to maintain a 5% false discovery rate. The adj.*p* values report the *p* values for a test for regulation (against an interval null of ±10%), adjusted to maintain a 5% false discovery rate. Note that where the adjusted *p* value is 1, the corresponding adjusted margin of error cannot be calculated and is listed as NA. Finally, the MoE columns report the raw 95% margins of error. Download Table 1-1, XLSX file.

**Figure 4.**
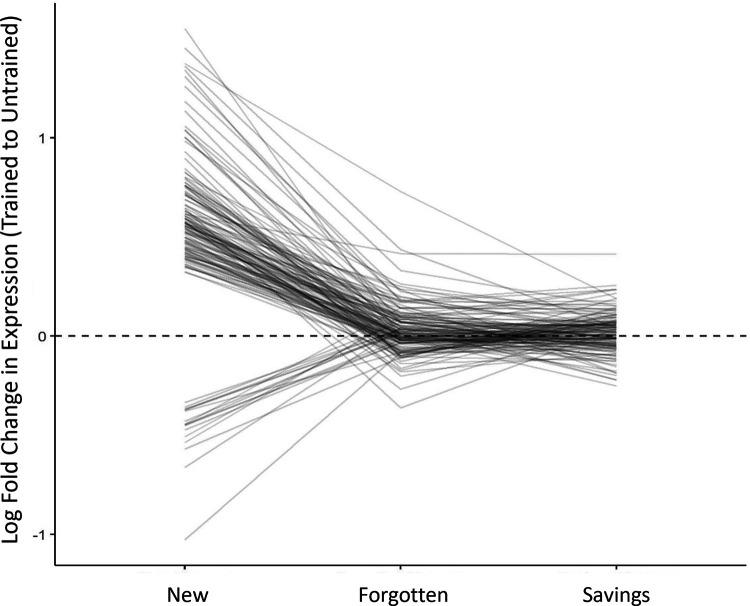
Fate of transcripts regulated during initial learning. This graph shows mean LFC (trained vs untrained) for each of the 148 transcripts flagged as clearly regulated in the new-memory group, tracking their expression during forgetting and savings. The dashed line at 0 represents no change in expression (when trained and untrained expression are the same, their ratio is 1, which gives an LFC of 0). Although these transcripts were clearly regulated 1 d after sensitization (new memory), none were clearly regulated during forgetting or savings.

In the new-memory group, there were 148 transcripts that were clearly regulated. The LFC for each of these transcripts is plotted by condition in Figure 4. As expected, nearly all these transcripts had been previously linked with the maintenance phase of sensitization memory (Liu et al., 1997; Conte et al., 2017) and represented predicted proteins with diverse functions, including signaling (ApTBL-1, GenBank: U57369.1, LFC =* *0.45 95% CI [0.09, 0.79]), protein production (an eIF2 subunit, GenBank: EB232654.1; LFC =* *0.57 95% CI [0.40, 0.74]), the unfolded protein response (GCN-1 like, GenBank: EB230807.1, LFC =* *0.53 95% CI [0.34, 0.72]), cytoskeletal function (septin-7-like, GenBank: EB260579.1; LFC =* *0.58 95% CI [0.29, 0.87]), and transport (a Dynein β chain component, GenBank: GD206216.1, LFC = −0.37 95% CI [−0.63, −0.12). Flagged transcripts also correctly included the positive control, ApBiP (LFC =* *0.79 95% CI [0.43, 1.16]).

Transcriptional regulation dissipated over time, with no transcripts flagged as clearly regulated in the forgotten-memory condition. This can be seen in Figure 4, where most transcripts regulated in the new-memory condition collapsed toward 0 in the forgotten-memory condition. Thus, defined in terms of overlapping regulation, the forgotten and new phases of memory showed no similarity (Table 1, column showing proportion of overlap). This was expected, as previous studies have also shown that the transcriptional response to sensitization training mostly fades over time and that the very few transcriptional changes that persist are difficult to detect with an array-wide screen (Patel et al., 2018; Perez et al., 2018). Indeed, although we had confirmed upregulation of FMRFa via qPCR is these samples (see Materials and Methods), for the microarray analysis this transcript did not meet the threshold for being flagged as clearly regulated (LFC =* *0.41 95% CI [0.19, 0.72], *p *=* *0.02 before correction, but *p *=* *1 after correction).

The critical question was if reactivating the memory during savings would reinstate the transcriptional regulation observed with a new memory. It did not. In the savings-memory group, there were also no transcripts flagged as clearly regulated. This missed at least some transcripts, as even the positive control, FMRFa, did not make the cutoff (LFC =* *0.67 95% CI [0.48, 0.86], *p *=* *0.0001 before correction, but *p *=* *1 after correction). Still, this indicates that among unequivocally regulated transcripts there was no overlap with the new-memory group. In Figure 4, this is shown as the lack of perturbation from the forgetting to the savings conditions. Thus, on the basis of this (admittedly crude) measure of similarity, the savings condition produced no reactivation of transcription, a finding that supports the retrieval-failure account of forgetting.

This was not because of an inability to detect similarity with this approach, as we could detect strong overlap between samples given similar treatments. Specifically, 141 of the 148 transcripts clearly regulated in the new-memory group were also flagged as clearly regulated in the archival new-memory group, which had also been harvested 1 d after sensitization training (Conte et al., 2017).

We also conducted a formal condition by training interaction analysis but did not find any transcripts showing a statistically significant change in training effect from the forgotten to savings stages of memory.

### How similar is savings memory to new memory? Gene ranking approach

Overlap of gene lists is not always a sensitive measure of similarity, as it depends on somewhat arbitrary significance classifications. Indeed, our stringent criteria clearly did not capture all regulated transcripts.

As a more sensitive way to measure similarity in transcriptional states, we compared rankings of transcripts across conditions using the using the OrderedList package in R (Yang et al., 2019). This allowed us to evaluate similarity in gene rankings across the 1000 most upregulated and downregulated transcripts in each condition regardless of statistical significance status. We compared the transcriptional state in the new-memory group to the forgotten-memory and savings-memory groups and benchmarked against the archival new-memory group.

**Figure 5.**
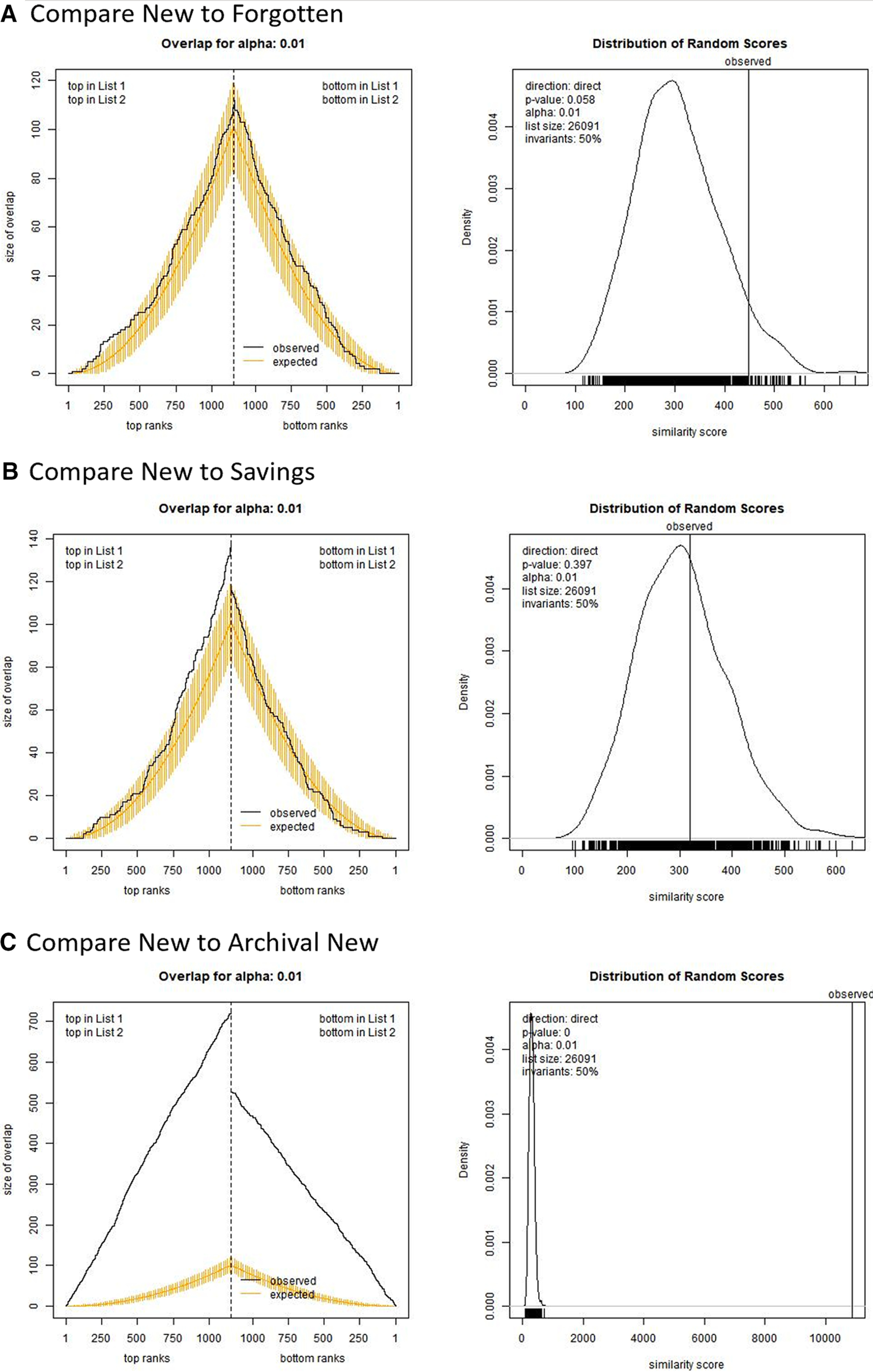
Similarity of conditions measured by rank-ordering of transcripts. These figures show similarity in the transcriptional states between the new-memory group and the forgotten-memory group (***A***), the savings-memory group (***B***), and the archival new-memory group (***C***). Panels on left compare observed similarity by gene-list length (black line) relative to the range of similarity observed with random shuffles of gene lists (yellow bars). Panels on right show overall similarity score for top ∼1000 genes against the distribution of scores from random shuffles.

Analysis of similarity by ranking also indicated that transcriptional regulation fades as a sensitization memory is forgotten. Comparing the new-memory to the forgotten-memory conditions showed only very weak similarity. Figure 5*A*, left, shows the observed levels of similarity relative to what is expected based on a random shuffle of gene lists. As can be seen, similarity tracks only slightly higher than the average expected by chance. Figure 5*A*, right, then compares the similarity score observed with the distribution of scores created with the random shuffles. This shows that the level of similarity is marginal (similarity score = 449.0, *p *=* *0.06). Thus, as forgetting progresses, the pattern of regulation shown during encoding is largely lost (although perhaps not completely).

Again, our critical question is what happens as sensitization memory is re-expressed via savings? Figure 5*B* shows this result, comparing the new-memory and savings groups. The savings group actually showed weaker similarity to the new-memory group (similarity score = 319.9, *p *=* *0.41). Note that although the scale of the similarity scores is arbitrary, it can be meaningfully compared across these analyses. Based on gene rankings, savings does not seem to appreciably reactivate the pattern of regulation observed as a memory is stored, a result more consistent with the retrieval-failure account of forgetting. Again, this was not because of a lack of sensitivity of our approach, as we could detect similarity between samples treated with similar protocols. Specifically, Figure 5*C* compares the new-memory group with the archival new-memory group; this shows very strong similarity in gene list rankings (similarity score = 10,873, *p *<* *0.0001).

### How similar is savings-memory to new memory? Correlational approach

One weakness of quantifying similarity based on rankings is that it omits the magnitude of regulation in the calculation of similarity. Thus, we made a third and final set of similarity measurements by examining correlations between changes in gene expression across conditions. Because we anticipated this to be the most sensitive and complete measure of sensitivity, we preregistered this measure as our primary outcome.

To calculate correlations, we first subsetted to the 148 transcripts clearly regulated in the new-memory group. We then examined the correlation in LFCs in these transcripts in the new-memory and savings-memory groups. We did this using a direct linear correlation (Pearson’s *r*) and with correction for possible measurement error using the genuine association (genas) function in the limma package for R.

**Figure 6.**
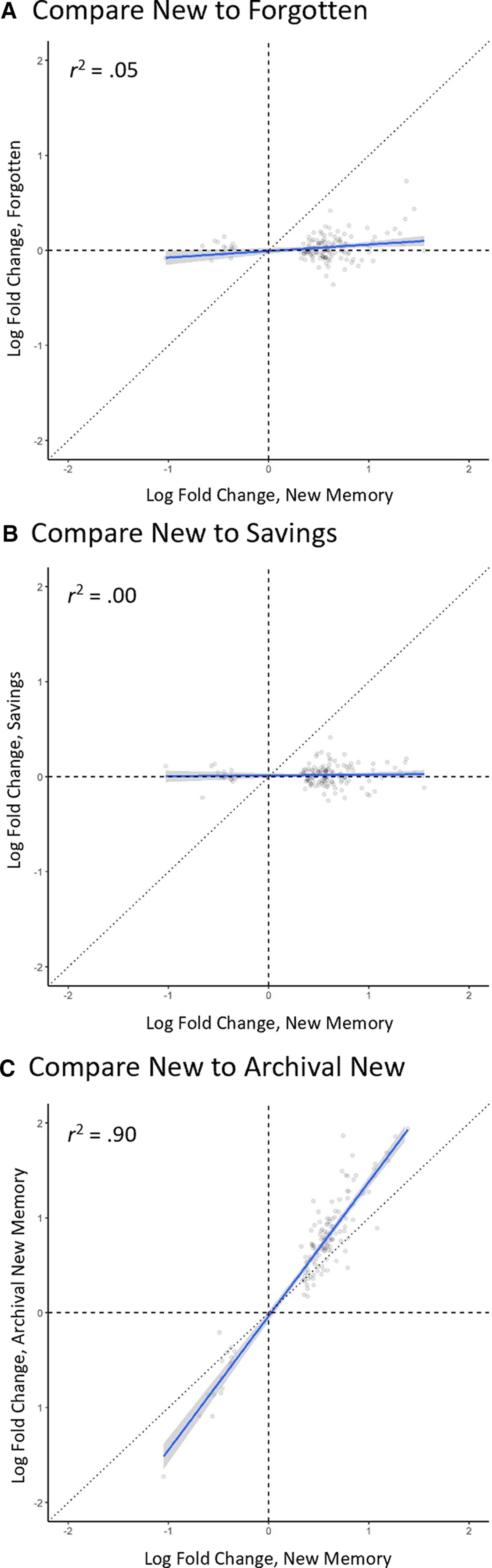
Similarity of conditions measured by correlation in LFCs. These figures show the correlation in LFC between the new-memory group and the forgotten-memory group (***A***), the savings-memory group (***B***), and the archival new-memory group (***C***). Only the 148 transcripts clearly regulated in the new-memory group are shown. The black dots are individual transcripts; the blue line is the line of best fit with shading indicating the 95% CI; the diagonal line shows a 1:1 relationship that would occur with perfect similarity.

Measuring similarity via correlation also indicated that the transcriptional changes that accompany encoding are largely (but not entirely) dissolved as forgetting proceeds: *r *=* *0.23 95% CI [0.07, 0.38]; *r*_corrected_ = 0.31, *p *=* *0.03 (Fig. 6*A*). These data are compatible with weak to modest levels of shared variance.

The critical question was if savings would reactivate encoding-related transcriptional changes. By this metric, the answer was again no. The correlation in regulation between the new and savings conditions was very weak when calculated on raw data (*r *=* *0.04 95% CI [–0.13, 0.20]; Fig. 6*B*) and only marginal when corrected for possible measurement error (*r*_corrected_ = 0.36, *p *=* *0.09). Calculated in raw scores, there was actually a decrease in correlation strength from forgetting to savings (*r*_savings-forgotten_ = –0.19, *p *=* *0.05). Calculated with correction for measurement error, there was a negligible increase in correlation strength (*r*_savings-forgotten_ = 0.05, *p *=* *0.30). This finding is most consistent with forgetting as retrieval failure. This was not because of lack of sensitivity, as we observed a strong correlation among samples treated similarly. Specifically, the correlation between the new-memory and archival new-memory groups was *r *=* *0.95 95% CI [0.93, 0.96] from raw scores and even higher (*r*_corrected_ = 0.99, *p *<* *0.001) when corrected for possible measurement error (Fig. 6*C*).

### Exploratory analyses

#### Comparison with previous results

We had previously characterized changes in gene expression that occur 1 d after sensitization training, identifying 1259 clearly regulated transcripts (the archival new-memory group drawn from Conte et al., 2017). We explored the degree to which these transcripts were similarly regulated in this new study. Specifically, we tested for regulation of just these putative memory-related transcripts in the new-memory condition, which was also harvested 1 d after sensitization training (although with additional pre-testing and sham training). We considered transcripts similarly regulated if they showed a statistically significant change in expression (null of no change), with correction to maintain a 5% false discovery rate.

We found strong consistency of results, with 77% of previously-identified transcripts qualifying as regulated in this focused test (972 of the 1259). Moreover, there was a strong correlation in regulation (*r *=* *0.93 95% CI [0.92, 0.94] across LFC scores, *N *=* *1258). This is consistent with the planned analyses reported above, which showed high similarity between these datasets. It is notable, however, that for the current study only 148 of these transcripts were flagged when screened over the whole array. Part of this discrepancy is because of the aggressive corrections required for a larger screen (whole array of 26,091 transcripts vs a focused test of 1198 transcripts). In addition, we noticed a general increase in within-group variation (noise) in the current study. Specifically, 70% of the transcripts identified in the archival new-memory group showed higher variance in the current study (larger standard deviation in LFC across samples). Overall, variance increased by 30% 95% CI [27%, 34%], raising thresholds for flagging a transcript as clearly regulated. This increase in noise did not seem to be because of an outlier or bad microarray sample (see next section). It may have been because of the longer duration of the protocol in the current study, which included sham training and three additional rounds of behavioral measurements (compare new-memory to archival new-memory protocols in Fig. 2). Consistent with this possibility, there was modest habituation evident in these animals just before training (see Fig. 3).

**Figure 7.**
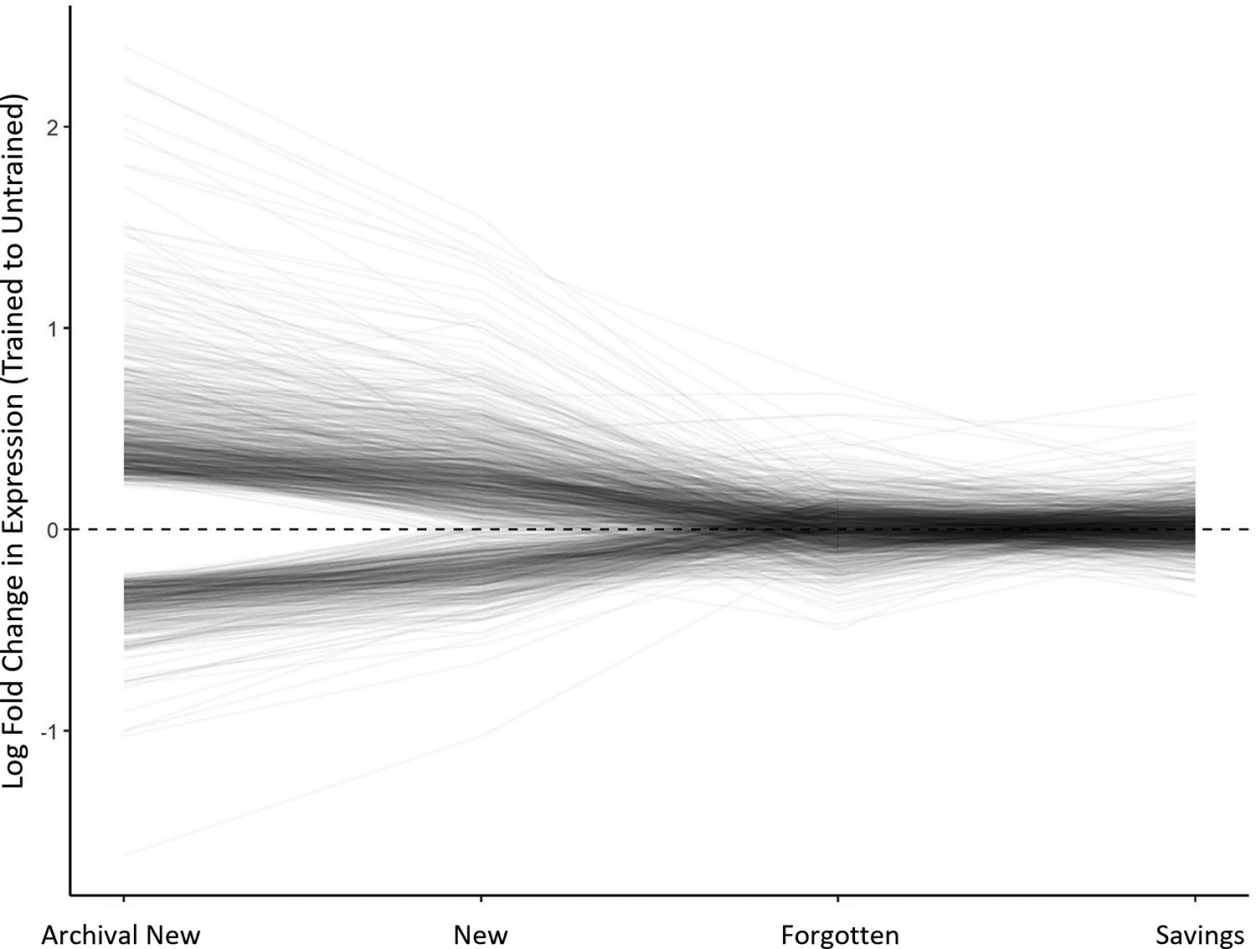
Exploratory analysis of fate of transcripts previously identified as regulated 1 d after sensitization training. This graph shows mean LFC (trained vs untrained) for 1198 transcripts which showed clear regulation in the archival new-memory condition (Conte et al., 2017), tracking their expression in the current new, forgetting, and savings groups. The dashed line at 0 represents no change in expression. Comparing the archival new-memory group to the current new-memory group shows some regression to the mean but that most transcripts show similar regulation. These transcriptional changes fade during forgetting (most transcripts collapse toward 0), and there is no obvious perturbation in expression when savings memory is induced.

Figure 7 shows the fate of the transcripts flagged in the archival new-memory group, plotting LFCs in the new, forgotten, and savings conditions. This graphically shows the strong consistency of expression between the archival and current new-memory conditions. Even with this broader set of transcripts, it is clear that regulation collapses during forgetting (most transcripts decline toward 0 in the forgotten-memory condition) and that savings does not strongly perturb gene expression (most transcripts remain near 0 in the savings-memory condition).

We have also previously examined changes in gene expression 7 d after sensitization training (Patel et al., 2018; Perez et al., 2018), finding via microarray and qPCR that there is persistent regulation in seven transcripts, all of which are also regulated during initial memory maintenance (1 d after training). To explore consistency with these previous results we tested for regulation just in these transcripts in both the forgotten-memory and savings-memory conditions (both of which were harvested 8 d after training). For this analysis we considered a transcript to be consistently regulated if there was a statistically significant change in expression (null of no change) with correction for multiple comparisons. Table 2 summarizes the results and compares them with previous findings. Overall, there was fair consistency, with four of seven transcripts showing regulation in the forgotten-memory condition, and two of seven in the savings-memory condition. None of these had been flagged in the array-wide screen reported above because of the lower-power of this analysis, but these focused tests again confirm that some transcriptional changes persist beyond the behavioral expression of sensitization memory. This focused test did not, however, reveal a strong change in regulation with savings memory.

**Table 2:** Transcriptional changes after forgetting of sensitization

Transcript	Description	Previous result	Forgetting	Savings
Z15041.1	ApBiP	0.63 ± 0.46	−0.04 ± 0.71	0.07 ± 0.31
M11283.1	FMRFa	0.53 ± 0.51	0.41 ± 0.26	0.67 ± 0.18
EB257711.1	LOC101857556	0.52 ± 0.46	0.26 ± 0.24	0.11 ± 0.32
EB254334.1	Transcribed locus	0.51 ± 0.50	0.73 ± 0.31	0.19 ± 0.58
FF066943.1	LOC106013098	0.36 ± 0.42	0.57 ± 0.13	0.49 ± 0.14
EB243511.1	Transcribed locus	−0.23 ± 0.50	0.00 ± 0.22	−0.21 ± 0.24
EB342172.1	Transcribed locus	−0.62 ± 0.35	−0.17 ± 0.23	0.08 ± 0.25

This table shows results for a set of seven transcripts previously identified via microarray and qPCR as being regulated after forgetting of sensitization. The previous result column is taken from Patel et al. (2018); it is qPCR data from pleural ganglia harvested 7 d after training, when sensitization had been forgotten. The forgetting and savings columns microarrays are from the current study, both of which were harvested 8 d after training. Numeric results are LFCs in expression ± the 95% margin of error (expanded for multiple comparisons).

### How robust are results to analysis parameters?

As the above considerations make clear, microarray results can depend critically on how the analysis is conducted. Although our plan was vetted through peer review and preregistered, it still represents only one reasonable analysis approach out of many. Thus, to examine the analysis generalizability of our results we conducted an exploratory multiverse analysis.

(1) To check for outsized influence of outliers we varied the inclusion of each the 32 microarray samples.

(2) As the new-memory group formed the focal point for comparisons, we ensured results would generalize by varying whether comparisons were made to the new-memory group or to the archival new-memory group.

(3) To ensure our results were not incomplete due to our discovery-based microarray approach we ran the analysis not only broadly on all 26091 unique transcripts on the array but also narrowly on 1198 transcripts we have previously identified as regulated by sensitization.

(4) Finally, we varied the stringency with which we flagged regulated transcripts, varying both the use of corrections for multiple comparison and the stringency of the null hypothesis (interval null of ±10% vs a standard null of no change).

These variations provided 528 different analysis specifications. We examined how results varied across these different analysis specifications.

Similarity based on overlap consistently supported forgetting as retrieval failure. The proportion of overlap between savings and the new-memory conditions was always modest, ranging from 0 up to 0.12. Moreover, overlap was always similar to or weaker than in the forgotten-memory conditions: *P*_diff_ ≤ 0.006 in all specifications.

Similarity based on ordered lists was also very stable, with no specifications indicating a statistically significant positive association in regulation between the new and savings phases of memory.

Similarity based on correlation also consistently supported forgetting as retrieval failure. The correlation in regulation between the savings-memory and new-memory condition was never more than moderate (*r* values ranged from –0.15 to 0.26). Moreover, the relationship was never substantially stronger than what was observed at forgetting: *r*_savings-forgotten_ was negative in 319 of 528 analyses, between 0 and 0.1 in 200 analyses, and between 0.1 and 0.21 in nine analyses. Thus, no analysis specification yielded an increase in similarity that met our preregistered criteria of at least 0.25 to indicate modest support for forgetting as decay.

Finally, leaving out each sample did not reveal any outlier that produced a strong or consistent influence on measures of similarity. In particular, we examined the effect of dropping the one sample from the forgotten-memory condition which had shown some habituation one week after training. This did not systematically alter any of the similarity measurements. Overall, our multiverse analysis suggests that our findings are robust to a variety of reasonable analysis specifications.

## Discussion

Sensitization training produces complex waves of transcriptional change. This transcriptional response is essential for creating a long-term memory, as blocking transcription during training abolishes long-term memory (Sutton et al., 2001), the long-term facilitation that mediates sensitization (Montarolo et al., 1986; Castellucci et al., 1989), and the structural correlates of long-term memory (Bailey et al., 1992) Moreover, several of the transcripts initially activated by sensitization training are essential for inducing the long-term facilitation thought to contribute to the behavioral expression of sensitization (e.g., C/EBP: Alberini et al., 1994; CREB1: Dash et al., 1990). This does not establish that transcriptional changes maintain long-term sensitization memory, but it is clear that transcriptional changes are required to activate maintenance mechanisms.

As sensitization is forgotten, the transcriptional changes produced by training also fade away. Here, we find that sensitization memory can be persistently reactivated (savings memory) without reactivating storage-related transcriptional changes. Savings memory seems mechanistically distinct from encoding a new memory, few transcripts are coregulated at both phases of memory and overall transcriptional states show only negligible similarity.

One limitation of this study is that power to detect clearly regulated transcripts was lower than anticipated. This means that our finding of no transcriptional changes during savings is tenuous; savings may activate some transcriptional changes that were missed.

This shortcoming did not limit our ability to compare transcriptional states. First, we planned for assessments of similarity based on global patterns of regulation, not just on the statistical-significance status of transcripts. We found that our measures of similarity could detect when samples had been treated alike, always showing very strong similarity between the new memory and archival new-memory conditions. In addition, our findings are robust to a range of reasonable analysis specifications, including ones which more aggressively classify transcripts as regulated.

Overall, our results are not consistent with decay models of forgetting. If the sensitization memory trace substantially decays during forgetting, savings would require at least partly rebuilding it. We found no evidence for this: long-term savings does not reactivate the transcriptional changes observed with an initial long-term memory. This suggests that the memory trace for sensitization remains reasonably intact and that forgetting of sensitization in *Aplysia* is because of retrieval failure.

This conclusion may seem puzzling: how could the memory trace be intact if there are almost no transcriptional changes that persist during forgetting and savings? The answer may be that the memory trace is molecularly sparse. For example, a recent screen found only two clear transcriptional changes in the hippocampus one week after fear conditioning, a time point when behavioral expression of the memory would still be quite strong (Mizuno et al., 2020). Thus, although learning initially produces widespread transcriptional changes, these may be refined to a relatively small core for maintenance. It is also possible that the maintenance mechanisms are not transcriptional at all, but merely require transcription to be initiated. For example, one (controversial) possibility is that the memory trace for sensitization is epigenetic, and requires transcriptional changes only for behavioral expression (Pearce et al., 2017). We are now working to determine whether the long-term expression of savings requires any transcriptional changes by injecting a transcriptional inhibitor just before the reminder shock used to evoke savings.

In retrieval-failure accounts of forgetting, memory traces persist yet gradually become decoupled from behavior. How might that happen in long-term sensitization? We have previously found that the transcriptional response to sensitization includes not only changes likely to promote memory but also changes likely to limit its behavioral expression. Specifically, we have found that sensitization training produces a robust and very long-lasting increase in the mRNA encoding FMRFa (Conte et al., 2017; Patel et al., 2018). In *Aplysia* FMRFamide is an inhibitory neuromodulator. It inhibits the VC neurons that help encode sensitization memory, depresses synapses, and decreases the strength of the T-SWR response (Abrams et al., 1984; Fioravante et al., 2006). This suggests the possibility that sensitization training not only builds the memory trace but also promotes inhibitory processes that can impair retrieval, a form of active forgetting (Davis and Zhong, 2017). If this is correct, it should be possible to manipulate the forgetting of sensitization memory by altering FMRFa signaling; we are now working to test this hypothesis.

Retrieval-failure accounts of forgetting propose that the memory trace is enduring, but that accessibility to retrieval is highly dynamic. Learning is thought to produce an initially accessible memory, time and new learning then decrease accessibility, but a variety of experiences can increase accessibility for short or long durations (context re-exposure, brief re-training, reminders, etc.). These different dynamics suggest that storage and retrieval are organized at different levels of neuronal function. Multiple lines of research indicate that encoding a long-term memory activates at least two distinct storage mechanisms: changes in synaptic efficacy and changes in connectivity. For example, in *Aplysia* long-term sensitization produces morphologic changes related to increased synaptic strength (increased active zone size and vesicle complement) and additional changes related to increased connectivity, such as new synaptic varicosities and active zones (Bailey and Chen, 1988). These decay at different rates, with changes in synaptic strength decaying over time while changes in connectivity endure (Bailey and Chen, 1989). In the mammalian visual system, changes in connectivity have specifically been shown to persist beyond forgetting (Linkenhoker et al., 2005; Hofer et al., 2009). One intriguing possibility, then, is that the memory trace is represented by enduring changes in connectivity while retrieval is made possible by more labile changes in synaptic strength. One testable prediction of this dual-process account of memory expression is that a generalized increase in activity could re-potentiate synapses and re-express a seemingly forgotten sensitization memory.

One critical question is how forgetting is related to other forms of memory disruption, such as the amnesia induced by disruptions of consolidation or reconsolidation. At the behavioral level, there are strong similarities, including the fact that memories which seem lost because of an amnestic intervention can often be re-expressed through a reminder or a brief retraining (Riccio and Richardson, 1984). On the other hand, current evidence indicates induced amnesia may be because of storage failure (Haubrich et al., 2020). Amnestic agents tend to degrade the physical correlates of memory. In contrast to our findings with forgetting, memory recovery after an amnestic agent tends to refresh the physical correlates of memory (Haubrich et al., 2020). Thus, forgetting and memory impairment through disrupted consolidation may be quite distinct. One difficulty with interpreting these results is that it is not yet possible to demarcate which brain correlates of memory are related to storage and which are related to accessibility. Still, it seems possible that induced amnesia and forgetting are mechanistically distinct. It would be useful to directly compare these processes in the same system.

Grand debates in science are never settled by one study. Our results strongly implicate retrieval failure as the mechanism of forgetting for sensitization in *Aplysia,* but a definitive account of forgetting will require additional studies across organisms and learning paradigms. The strategy of monitoring neuronal changes across multiple memory states seems likely to be fruitful for better resolving how and why forgetting occurs.

## Synthesis

Reviewing Editor: Christie Fowler, University of California, Irvine Decisions are customarily a result of the Reviewing Editor and the peer reviewers coming together and discussing their recommendations until a consensus is reached. When revisions are invited, a fact-based synthesis statement explaining their decision and outlining what is needed to prepare a revision will be listed below. The following reviewer(s) agreed to reveal their identity: Jonathan Ploski.

Both of the reviewers feel that the manuscript is of high importance for the field and interesting. It was also noted that the studies are well-executed, and the manuscript clearly conveys the main findings in a comprehensive manner. However, we agree that the discussion warrants additional consideration of limitations for interpretation of these data sets. Specifically, the manuscript would be significantly strengthened by explaining better the exact evidence for the role of transcription in this process and underscore any potential weaknesses or doubts regarding the role of transcription to avoid over-interpretation of these findings. Thus, please address the following 3 points in the discussion:

1. The validity of these conclusions for this study rest heavily on the notion that the transcripts that show a change in expression 1 day after training are critically involved in the encoding or maintenance of sensitization. However, this is not necessarily true and if they are not, then this would lead to the wrong conclusion. Therefore, please discuss what is the exact evidence that these “regulated” transcripts 1 day after sensitization training are indeed required to encode/maintain sensitization. Has any one of these transcripts been blocked during this time (1 day after sensitization training) and found that sensitization was lost? Does blocking transcription 1-5 days after training result in the lost of sensitization? However, if so, this isn’t entirely supportive because it can’t rule out that it’s not the differentially expressed transcripts that are important but rather some constitutively expressed transcript that when blocked perturbs sensitization expression. If these data have not been demonstrated, then please discuss these points as a limitation of the current study and indicate the need for future investigations.

2. The authors seem to imply that the memory could only exist due to an altered transcriptional state. But this isn’t necessarily true. Proteins with half lives longer than days could explain changes in sensitization, and/or constitutively active kinases etc. This should be more fully discussed.

3. Consolidation and reconsolidation are discussed, but there appears to be some important mechanistic differences between these processes and sensitization. For one, administering a transcriptional or translationally inhibitor 24 hours after memory encoding or retrieval would have no influence on the memory.
